# Magical manoeuvre: a 5-second instructor's intervention helps lightweight female rescuers achieve the required chest compression depth

**DOI:** 10.1186/cc14495

**Published:** 2015-03-16

**Authors:** A Krikscionaitiene, A Pranskunas, M Dambrauskiene, N Jasinskas, Z Dambrauskas, E Vaitkaitiene, J Vencloviene, D Vaitkaitis

**Affiliations:** 1 Lithuanian University of Health Sciences, Kaunas, Lithuania

## Introduction

Adequate chest compression (CC) depth is crucial for resuscitation outcomes. Lightweight rescuers, particularly women, are often unable to achieve the required 5 to 6 cm CC depth. This nonrandomised cohort study investigated new strategies to improve CC performance.

## Methods

Data were prospectively collected from January 2011 to January 2012 from 336 female medical and pharmacy students undergoing CPR training at the Lithuanian University of Health Sciences. During the training process, the instructors performed a simple 5-second intervention (Andrew's manoeuvre) with all of the rescuers in the study group. The instructor pushed 10 times on the shoulders of each trainee while she performed CCs to achieve the maximal required compression depth. Immediately after training, the participants were asked to perform a 6-minute BLS test on a manikin that was connected to a PC with SkillReporter™ System software (Laerdal, Norway); the quality of the participants' CPR skills was then evaluated.

## Results

The CC depth in the study group increased by 6.4 mm (*P *< 0.001) compared with the control group (52.9 vs. 46.6 mm). A regression analysis showed that Andrew's manoeuvre increased the depth of the CCs among women by 14.87 × (1 - 0.01 × weight) mm.

## Conclusion

Andrew's manoeuvre during CPR training significantly improved the performance of the female rescuers and helped them achieve the CC depth required by 2010 resuscitation guidelines. It is most effective among the women with the lowest body weight.
